# Is craniofacial pain associated with mental health disorders in adults and older adults in Southern Brazil?

**DOI:** 10.1590/1807-3107bor-2026.vol40.075

**Published:** 2026-10-16

**Authors:** Antônio Augusto SCHÄFER, Micaela Rabelo QUADRA, Julia Niero Zanatta STRECK, Vanessa Caldardo Lemes da SILVA, Emilio Luiz STRECK, Marcelo Bighetti TONIOLLO, Fernanda Oliveira MELLER

**Affiliations:** (a) Graduate Program in Public Health, University of Southern Santa Catarina, Criciúma, Brazil. Medical School, University of Rio Verde, UniRV, Rio Verde, Brazil.; (b) Graduate Program in Health Sciences, University of Southern Santa Catarina, Criciúma, Brazil.; (c) Graduate Program in Health Sciences, University of Southern Santa Catarina, Criciúma, Brazil.; (d) Medical School, University of Southern Santa Catarina, Criciúma, Brazil.; (e) Graduate Program in Health Sciences, University of Southern Santa Catarina, Criciúma, Brazil. Medical School, University of Rio Verde, UniRV, Rio Verde, Brazil.; (f) Dental School of Rio Verde, University of Rio Verde (FORV/UniRV), Rio Verde, Brazil.; (g) Graduate Program in Public Health, University of Southern Santa Catarina, Criciúma, Brazil.

**Keywords:** Facial Pain, Depression, Stress, Stress, Psychological, Sex Factors, Cross-Sectional Studies

## Abstract

Chronic pain and psychiatric disorders are commonly present in the same person, and the interaction of these conditions can influence the intensity, duration, and frequency of the pain. Thus, this study aimed to examine the association between craniofacial pain and mental health disorders among individuals aged 18 years or older in Southern Brazil, using sex-stratified analyses. This cross-sectional population-based study was carried out in a city in Southern Brazil. Depressive symptoms and perceived stress were assessed by the Patient Health Questionnaire-9 and the Perceived Stress Scale-14. Jaw and temporal pain were self-reported. Crude and adjusted Poisson regression models were used to analyze the associations. In the 820 participants, the prevalence values of jaw and temporal pain, depression, and stress were 17.2%, 18.3%, 29.2%, and 35.8% respectively, all of them higher in females. After adjustments, temporal pain was associated with a higher prevalence of depressive symptoms in the total population (PR = 1.83; 95%CI: 1.43–2.33), males (PR = 2.46; 95%CI: 1.48–4.07), and females (PR = 1.69; 95%CI: 1.29–2.22). Temporal pain was also associated with stress in the total population (PR = 1.29; 95%CI: 1.05–1.60) and in females (PR = 1.46; 95%CI: 1.16–1.84). In conclusion, morning temporal pain was significantly associated with depressive symptoms in the total population and in both men and women, while for perceived stress this association was found only in the total population and in women.

## Introduction

Chronic pain and psychiatric disorders are frequently present in the same individual and may display overlapping symptoms.^1^ Both conditions have a high socioeconomic impact, negatively affect quality of life and well-being, and share accompanying factors, such as sleep impairment, physical disability, and psychosocial challenges.^1,2^

Among chronic pain disorders, headaches - particularly migraines and chronic daily headaches - stand out as some of the most prevalent. In 2019, an estimated 581.8 million migraine cases were reported around the world. Moreover, a recent review indicated a global prevalence of 52.0% for headache, 14.0% for migraine, and 26.0% for tension-type headache.^3^ In Brazil, a cross-sectional study found a prevalence of 40.3% for tension-type headache and 29.2% for migraine.^4^

The strong interactions between headaches and depression, stress, and anxiety can influence the intensity, durability, frequency, and impact of pain.^5^ Bidirectionally, more frequent pain may lead to the presence of a depressive or anxiety state, aggravating existing disorders or acting as a trigger to initiate a psychiatric condition.^6,7^ In agreement, studies show that pain and psychiatric disorders share biological mechanisms and pathways, where chronic pain may lead to psychiatric disorders or vice versa, through neural changes or activations that occur in both conditions.^8^

Pain in the temporal and masseter muscles is central to orofacial pain and temporomandibular disorders (TMD), often contributing to chronic facial pain. This pain is also influenced by psychological factors.^9,10^ Specifically, morning headaches have been reported to affect around 5 to 8% of the population, and this may reach up to 33% in patients with obstructive sleep apnea.^11,12^ Its occurrence is related to sleep problems, such as bruxism, and mood disorders.^13^

The relationship between sleep disorders, chronic pain, and psychiatric disorders is complex and interrelated, and few studies have evaluated the influence of confounding factors when analyzing morning craniofacial pain. Although previous studies have considered the confounding influence of demographic, psychosocial, and general health characteristics, they generally do not incorporate confounders related to sleep and orofacial function, or mental health outcomes, such as sleep quality, sleep bruxism, and obstructive sleep apnea.^5,6,12-16^ Moreover, sex differences have been reported in the literature regarding temporomandibular disorders and orofacial pain, with anatomical, hormonal, and neuromodulatory features highlighted as accounting for the distinct responses observed.^17^

Thus, assessing the association between craniofacial pain and psychiatric comorbidities - while adjusting for sleep-related variables and considering possible sex differences - is crucial for comprehensive understanding and management of facial pain. In this study, craniofacial pain refers specifically to self-reported morning temporal pain and morning jaw pain, which differ from diagnostic categories such as headache or temporomandibular disorders cited in the previous literature. Accordingly, the current study used data from a population-based survey to examine the association between craniofacial pain and mental health disorders among individuals aged 18 years or older in Southern Brazil, using sex-stratified analyses.

## Methods

### Study design and sample

Data were collected from a population-based cross-sectional study conducted in the city of Criciúma, Brazil. Criciúma has 214,493 inhabitants and is located in the southern state of Santa Catarina, about 200 km from the state capital, Florianópolis.

The target population included individuals aged 18 years or older residing in the urban area of the city. Data collection was carried out between March and December 2019.

The sampling process was conducted in two stages, based on data from the 2010 Demographic Census: the primary units were the census tracts, and the secondary units were the households. Initially, all urban census tracts with private households were listed (n = 306) in the order of their sector codes. Subsequently, 25% (n = 77) of these census tracts were randomly selected, totaling 15,218 households. The number of households was sampled proportionally to the size of each sector; 618 households were then systematically selected within the chosen tracts for visits. The sample size was calculated considering a statistical power of 80%, 95% confidence interval, and 50% for unknown outcome prevalence. A sampling error of five percentage points was adopted, and to account for the complex sampling design, a design effect of 1.5 was applied. Additionally, an increment of 15% was included for potential losses and refusals. Based on these parameters, the minimum required sample was estimated at 662 individuals.

All individuals aged 18 years or older residing in the urban area of the city were eligible for the study. Exclusion criteria included individuals unable to respond to or complete the interview due to physical or cognitive impairments. However, no participants met the exclusion criteria during data collection.

### Data collection

The data were collected through face-to-face interviews using a single, standardized, pre-coded questionnaire. The instrument, which had an average administration time of 30 minutes, was applied by a team of 12 trained interviewers to participants who provided written informed consent. Before data collection, the interviewers participated in a structured training and calibration process that included theoretical instruction, standardized guidelines for administering each item, and supervised practice interviews to ensure consistency in questionnaire application. The data collected were reviewed, coded, and double-entered using EpiData 3.1 software.

### Studied variables

#### Mental health variables

The mental health disorders evaluated were depressive symptoms and perceived stress. Depressive symptoms were assessed by the *Patient Health Questionnaire-9* (PHQ-9), which has been validated for the Brazilian population. This tool investigates the frequency of depressive symptoms (depressive mood, anhedonia, trouble sleeping, tiredness or lack of energy, change in appetite or weight, feeling of guilt or uselessness, trouble concentrating, feeling slow or agitated, and thoughts about death or suicidal ideation) in the two weeks before the interview. Each of the 9 questions on the questionnaire is scored from 0 to 3, corresponding to “never”, “less than once per week”, “once per week or more”, and “almost every day”. The cut-off point of ≥ 9 proposed by Santos et al. ^18^ was adopted. Individuals who scored 9 or more in the test were considered as having positive screening for depressive symptoms.

Perceived stress was assessed using the *Perceived Stress Scale* (PSS-14), previously validated for the Brazilian population.^19^ The PSS-14 is a 14-item scale that assesses stressful experiences in the month before the interview. The scale has a possible final score ranging from 0 to 56 points. To define perceived stress in the current study, the score was categorized into quintiles, in which individuals classified in the fourth or fifth quintiles were considered as presenting symptoms of stress. Perceived stress has been defined by this approach in previous studies.^20-23^

Craniofacial pain variables

Craniofacial pain (morning jaw pain and morning temporal pain) were assessed through self-reported questions: “Do you feel tiredness or pain in the jaw or mouth muscles when you wake up?” and “Do you feel pain in your temples (the sides of your head, above the ears) when you wake up?”. The answer options were “yes” or “no”. In the regression models, both variables were included as nominal categorical variables, with “no” defined as the reference category. These measures reflect participants’ perceptions of craniofacial pain symptoms.

## Covariates

Sociodemographic variables were included as potential confounders of the relationship between craniofacial pain and mental health outcomes. The variables included were sex (male, female), age (collected in completed years and categorized as 18–29, 30–39, 40–49, 50–59, 60 or more), schooling (collected in completed years and categorized as 0–4, 5–8, 9–11, 12 or more), income (BRL per month) (< 500.00, 500.00–1,000.00, 1,001.00–2,000.00, 2,001.00–4,000.00, > 4,000.00), self-reported sleep bruxism (never, rarely, sometimes, frequently), self-reported sleep quality (very good, good, regular, poor, very poor), and self-reported obstructive sleep apnea (no, yes).

## Statistical analysis

Descriptive analyses of all variables were performed, and presented as absolute (n) and relative (%) frequencies and their respective 95% confidence intervals (95%CI). The prevalence of craniofacial pain and mental health variables was also stratified by sex.

Crude analysis of the association between craniofacial pain and mental health variables was performed using Poisson Regression, with a 5% significance level. Adjusted analyses were also performed to assess whether the associations between craniofacial pain and mental health were independent of possible confounders. Poisson Regression was used, and the results are reported as prevalence ratios (PR) and their corresponding 95%CI. Variables with a 20% significance level (p-value < 0.20) were considered confounders and remained in the final model.

Two hierarchical models were analyzed. Model 1 included sociodemographic and behavioral variables (age, schooling, sleep quality, sleep bruxism, and obstructive sleep apnea). Model 2 included all variables from Model 1 and additionally incorporated the two craniofacial pain variables with mutual adjustment: when morning jaw pain was the exposure variable, morning temporal pain was included as an adjustment variable; conversely, when morning temporal pain was the exposure, morning jaw pain was included as an adjustment variable. This structure ensures that the associations for each pain site are estimated independently of the others. All analyses were stratified by sex.

The statistics program Stata version 16.1 (https://www.stata.com) was used to perform all analyses using the svy prefix, which considers the complexity of the sampling process and the effect of the study design.

## Ethical considerations

The project was approved by the Research Ethics Committee of the University of Southern Santa Catarina in December 2018 under protocol number 3.084.521. All participants provided written informed consent. The study was conducted in full accordance with the Declaration of Helsinki.

## Results

A total of 820 individuals were interviewed (86.1% response rate). Females (63.8%) and older people (45%) made up most of the sample. More than half of the participants had 8 years of schooling or less (53.6%), and approximately 40% of them earned up to 1,000.00 BRL per month. Approximately one in every ten individuals reported frequent sleep bruxism (8.3%; 95%CI: 6.3–10.2) and obstructive sleep apnea (8.5%; 95%CI: 6.5–10.4), and about one fifth considered their sleep quality as very poor/poor (18.9%) (Table 1).

**Table 1:** Characteristics of adults aged 18 years or older in Criciúma, Santa Catarina, Brazil, 2019 (n = 820).

Variables	n	% (95%CI)
Sex		
Male	297	36.2 (32.9–39.5)
Female	523	63.8 (60.5–67.1)
Age (years)		
18–29	101	12.3 (10.1–14.6)
30–39	93	11.3 (9.2–13.5)
40–49	85	10.4 (8.3–12.5)
50–59	172	21.0 (18.2–23.8)
≥ 60	369	45.0 (41.6–48.4)
Schooling (years)		
0–4	219	26.7 (23.7–29.8)
5–8	220	26.9 (23.8–29.9)
9–11	266	32.5 (29.3–35.7)
≥12	114	13.9 (11.5–16.3)
Income (BRL per month)*		
< 500.00	151	19.0 (16.3–21.7)
500.00–1,000.00	166	20.9 (18.0–23.7)
1,001.00–2,000.00	248	31.2 (28.0–34.4)
2,001.00–4,000.00	164	20.6 (17.8–23.4)
> 4,000.00	66	8.3 (6.4–10.2)
Sleep bruxism		
Never	665	83.1 (80.5–85.7)
Rarely	25	3.1 (1.9–4.3)
Sometimes	44	5.5 (3.9–7.1)
Frequently	66	8.3 (6.3–10.2)
Sleep quality		
Very good	65	7.9 (6.1–9.8)
Good	360	43.9 (40.5–47.3)
Regular	232	28.3 (25.2–31.4)
Poor	111	13.5 (11.2–15.9)
Very poor	52	6.4 (4.7–8.0)
Obstructive sleep apnea		
No	734	91.5 (89.6–93.5)
Yes	68	8.5 (6.5–10.4)

SD: standard deviation. CI: confidence interval. *Variable with highest number of missing data points (n = 25)

The prevalence values of jaw and temporal pains were 17.2% (95%CI: 14.6–19.8) and 18.3% (95%CI: 15.7–21.0), respectively. Women had a greater frequency of both types of pain (20.6% of jaw pain and 23.4% of temporal pain) compared to men (11.2% of jaw pain and 9.4% of temporal pain). Depressive symptoms were observed in 29.2% of the sample– this frequency was higher among females when compared to males (35% vs. 18.8%). The prevalence of perceived stress was 35.8%, being 37.5% among women and 32.9% among men (Figure).

**Figure:**
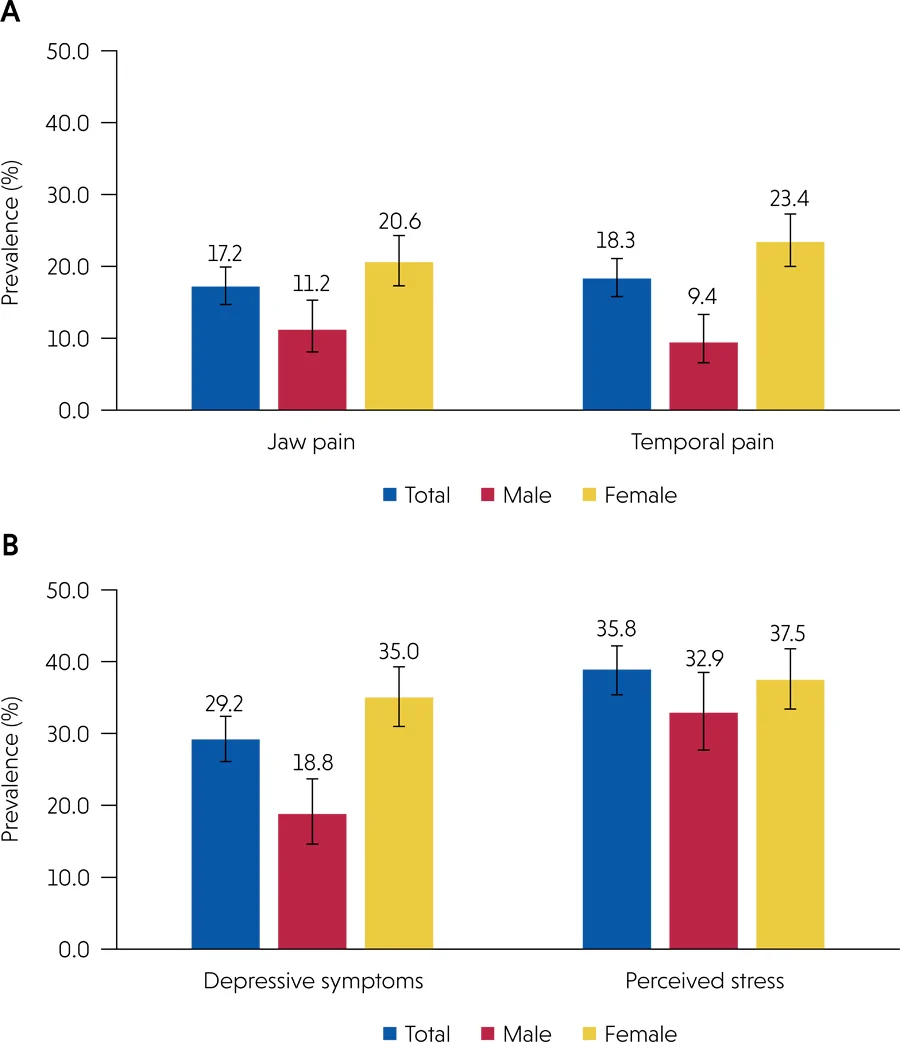
Prevalence of A) craniofacial pain and B) mental health symptoms in the total population and stratified by sex. Criciúma, Santa Catarina, Brazil, 2019. (n = 820)

The crude analysis showed that both morning jaw and temporal pains were associated with depression in the total sample and in both sexes. Stress was not associated with morning jaw pain, but it was related to morning temporal pain in the total sample and in females (Table 2).

**Table 2:** Crude analysis* of the association of craniofacial pain with depressive symptoms and perceived stress, for the total sample and stratified by sex. Criciúma, Santa Catarina, Brazil, 2019. (n = 820)

Variable	Depressive symptoms			Perceived stress		
	Total	Male	Female	Total	Male	Female
	PR (95%CI)	PR (95%CI)	PR (95%CI)	PR (95%CI)	PR (95%CI)	PR (95%CI)
Morning jaw pain	p<0.001	p = 0.008	p<0.001	p = 0.053	p = 0.369	p = 0.130
No	Reference	Reference	Reference	Reference	Reference	Reference
Yes	2.10 (1.71–2.60)	2.10 (1.22–3.64)	1.92 (1.53–2.40)	1.24 (1.00–1.55)	1.23 (0.78–1.96)	1.22 (0.94–1.57)
Morning temporal pain	p<0.001	p < 0.001	p < 0.001	p = 0.001	p = 0.620	p = 0.001
No	Reference	Reference	Reference	Reference	Reference	Reference
Yes	2.80 (2.31–3.40)	3.14 (1.93–5.12)	2.45 (1.98–3.03)	1.43 (1.17–1.75)	1.14 (0.68–1.92)	1.48 (1.18–1.86)

*Poisson regression. PR: prevalence ratio. CI: confidence interval.

In the adjusted analyses, Model 1 included sociodemographic and behavioral variables and Model 2 additionally incorporated mutual adjustments between the two craniofacial pain variables (i.e., temporal pain was included as a covariate when jaw pain was the exposure, and vice versa). After adjustment, morning temporal pain remained associated with depression in the total sample and in both sexes in both models. In model 2, men and women who reported morning temporal pain were, respectively, 2.46 (95%CI: 1.48–4.07) and 1.69 (95%CI: 1.29–2.22) times more likely to present depression than those without morning temporal pain.

In relation to morning jaw pain, in Model 1, the initial findings showed that this variable was associated with depression. However, after including morning temporal pain as a confounding variable in Model 2, the association disappeared, indicating an influence of temporal pain in this association (Table 3).

**Table 3:** Adjusted analysis* of the association between craniofacial pain and depressive symptoms, for the total sample and stratified by sex. Criciúma, Santa Catarina, Brazil, 2019 (n = 820).

Variable	Depressive symptoms					
	Model 1			Model 2		
	Total	Male	Female	Total	Male	Female
	PR (95%CI)	PR (95%CI)	PR (95%CI)	PR (95%CI)	PR (95%CI)	PR (95%CI)
Morning jaw pain	p < 0.001	p=0.028	p < 0.001	p=0.109	p=0.336	p=0.267
No	Reference	Reference	Reference	Reference	Reference	Reference
Yes	1.61 (1.31–1.99)	1.80 (1.06–3.04)	1.50 (1.20–1.88)	1.22 (0.96–1.55)	1.35 (0.73–2.48)	1.16 (0.89–1.50)
Morning temporal pain	p < 0.001	p < 0.001	p < 0.001	p < 0.001	p < 0.001	p < 0.001
No	Reference	Reference	Reference	Reference	Reference	Reference
Yes	1.99 (1.62–2.45)	2.46 (1.48–4.07)	1.85 (1.48–2.30)	1.83 (1.43–2.33)	2.46 (1.48–4.07)	1.69 (1.29–2.22)

*Poisson regression. PR: prevalence ratio. CI: confidence interval. Model 1: Adjusted for age, schooling, income, sleep quality, sleep bruxism, and obstructive sleep apnea. Model 2: Adjusted for all variables from Model 1 plus morning temporal pain (when morning jaw pain was the exposure variable) and morning jaw pain (when morning temporal pain was the exposure variable).

Morning jaw pain was not associated with stress, in either the crude or adjusted models. On the other hand, morning temporal pain was related to stress in the total sample and females in both adjusted models. In the total sample, individuals with morning temporal pain had a 29% higher prevalence of stress in both models (model 1: 95%CI: 1.04–1.60– model 2: 95%CI: 1.05–1.60). Among women, the association was 37% higher in Model 1 (95%CI: 1.09–1.73) and became stronger in Model 2 (PR: 1.46– 95%CI: 1.16–1.84), when morning jaw pain was included as a confounding variable.

## Discussion

The findings of the current study, using a population-based survey, provide novel insights into the relationship between craniofacial pain and mental health, identifying sex differences that can influence the clinical management and public health actions for these conditions. While reported morning temporal pain was associated with a higher prevalence of depressive symptoms in the total population and in both sexes, its association with stress was observed only in the total sample and among women. In contrast, in the final model, morning jaw pain was not related to any mental health symptoms in any of the stratified analyses.

Before discussing the associations, it is important to highlight the prevalence of mental health symptoms and pain disorders among the participants of this study: both depressive symptoms and perceived stress, as well as jaw and temporal pain, were more prevalent in women than in men. Previous studies agree with these findings. In South Korea, a study with individuals with TMD observed that the risk for depressive symptoms was 2.08 times higher for women than men.^24^ Another survey with dental students showed that 54.7% reported stress, with 20.2% reporting severe results. Moreover, it was observed that stress levels were lower among male students^25^ (Table 4)

**Table 4:** Adjusted analysis* of the association between craniofacial pain and perceived stress, for the total and stratified by sex. Criciúma, Santa Catarina, Brazil, 2019 (n = 820).

Perceived stress						
Variables	Model 1			Model 2		
	Total	Male	Female	Total	Male	Female
	PR (95%CI)	PR (95%CI)	PR (95%CI)	PR (95%CI)	PR (95%CI)	PR (95%CI)
Morning jaw pain	p = 0.217	p = 0.475	p = 0.288	p = 0.949	p = 0.475	p = 0.706
No	Reference	Reference	Reference	Reference	Reference	Reference
Yes	1.15 (0.92–1.43)	1.18 (0.75–1.85)	1.14 (0.89–1.46)	1.01 (0.77–1.31)	1.18 (0.75–1.85)	0.94 (0.70–1.27)
Morning temporal pain	p = 0.018	p = 0.780	p = 0.007	p = 0.018	p = 0.780	p = 0.001
No	Reference	Reference	Reference	Reference	Reference	Reference
Yes	1.29 (1.04–1.60)	1.08 (0.63–1.83)	1.37 (1.09–1.73)	1.29 (1.05–1.60)	1.08 (0.63–1.83)	1.46 (1.16–1.84)

*Poisson regression. PR: prevalence ratio. CI: confidence interval. Model 1: Adjusted for sex, age, schooling, income, sleep quality, sleep bruxism, and obstructive sleep apnea. Model 2: Adjusted for all variables from Model 1 plus morning temporal pain (when morning jaw pain was the exposure variable) and morning jaw pain (when morning temporal pain was the exposure variable).

Mental health disorders have shown a consistent global increase. The age-standardized incidence rate of depression reached 3,588.25 per 100,000 inhabitants in 2019, with considerably higher rates among women.^26^ Stress was reported to affect more than one-third of the global population, being more common in low- and middle-income countries and among women.^27^ Depression is known to not only influence the course of TMD but also to exacerbate pain.^16^ Stress, in turn, is characterized by a state in which homeostasis is threatened, triggering changes in the central nervous system that are essential for the fight-or-flight response.^28^ A systematic review and meta-analysis indicated that 43% of patients with TMD present depression, which can worsen the clinical condition.^29^ Furthermore, the chronicity of TMD in up to 30% of cases may intensify its psychological impact^30^

According to a systematic review and meta-analysis of population-based studies, the prevalence of TMD is higher in women than in men, with women presenting a 2.24-fold increased risk for global TMD.^31^ In the Brazilian population, the same pattern is observed: the prevalence of TMD in the total population is around 33%, and the prevalence is also higher in women (37%) than in men (29.3).^32^ This pattern of a higher prevalence in women, both for pain and mental health disorders, suggests the influence of neuroendocrine and psychosocial factors.^17^

To discuss the results of the association between craniofacial pain and mental health, it is first important to establish that TMD is a musculoskeletal condition responsible for impairing the function of the temporomandibular joint (TMJ) and masticatory muscles, leading to pain, headache, and restricted mouth opening, and it may also be linked to psychological and dental factors.^33,34^ Multiple risk factors have been identified for TMD, among them: female sex, mental health symptoms, sleep disorders, comorbidities such as headaches (various types) and irritable bowel syndrome, pain perception, and jaw mobility.^35^

The association between morning temporal pain and depressive symptoms, even after adjusting for sociodemographic and clinical variables, in the total population and stratified by sex, suggests a specific relationship between psychological distress and muscle tension or sensitivity in the temporal region. These findings support the integration of mental health assessments into the clinical management of chronic orofacial pain, particularly in cases involving temporal muscles.

On the other hand, after adjustment for potential confounders, morning temporal pain was associated with stress only in the total population and in the female sex. Psychological stress has been described in the literature as being associated with increased muscle tone, particularly in the temporal muscle, often involved in stress-related dysfunctional habits, such as daytime clenching.^36^ Women may be more vulnerable to stress due to work and family responsibilities.^37^ Furthermore, the presence of estrogen receptors in the TMJ has been linked to inflammation and joint degeneration in women, and estrogen has been associated with increased cartilage and bone joint degradation.^38^ In the present study, given the cross-sectional design, it is not possible to determine whether mental health symptoms contributed to increased muscle tension or pain perception, whether craniofacial pain exacerbates mental health symptoms, or whether both processes occur bidirectionally.

Finally, the lack of association after adjustments between morning jaw pain and mental health disorders reinforces the need for differentiated analysis between affected anatomical regions and raises the hypothesis of region-specific sensitivity of craniofacial structures to emotional states. Therefore, the data presented here underscore the importance of distinguishing between different types of facial pain, since morning temporal pain, often related to overload of the temporal muscles and myofascial dysfunction, may be more sensitive to the influence of negative emotional states than morning jaw pain.

Regarding the limitations of the current study, the cross-sectional design must be highlighted as it does not allow the establishment of causality of the findings. Therefore, it is not possible to determine whether craniofacial pain contributes to mental health symptoms or whether mental health symptoms increase the perception or reporting of pain. In addition, morning jaw and temporal pain, sleep bruxism, and obstructive sleep apnea were self-reported at the time of the interview, without clinical examination or objective diagnostic confirmation, which may affect prevalence estimates. As a result, these variables are subject to recall and misclassification bias, leading to potential under- or overestimation of their frequency and affecting the estimated associations. Depressive symptoms and perceived stress were assessed using screening tools rather than diagnostic instruments– however, as mentioned in the methods section, the PHQ-9 and PSS are commonly used for population-based studies. In addition, there are no universally established cut-off points for identifying stress levels for the PSS, thus, we chose quintiles as a sample-based strategy to distinguish participants according to the relative distribution of perceived stress. Moreover, the assessment of morning jaw pain was based on a question including both muscle tiredness and pain, and considering that these symptoms differ clinically, grouping them may have introduced bias, leading to potential overestimation of the prevalence of jaw pain. Finally, although we used a robust hierarchical adjustment model, including sociodemographic and clinical confounders, residual confounding could still exist.

As for strengths, the current study provides valuable insights into the assessment of craniofacial pain and its association with mental health disorders using data from a population-based survey, with a robust, two-stage sampling process, ensuring a representative sample of adults and older individuals. Additionally, the analyses stratified by sex allowed the exploration of potential differences in associations, contributing to better understanding of the role of sex in craniofacial pain and mental health outcomes. Moreover, although the study involved a lengthy questionnaire with an average administration time of 30 minutes, strategies were implemented to maintain participant engagement throughout the interview. These strategies included interviewer training and the use of standardized procedures, which ensured that participants remained comfortable and motivated during data collection.

## Conclusion

In conclusion, morning temporal pain was significantly associated with depressive symptoms in the total population as well as in both men and women, while for perceived stress this association was only found in the total population and in women. The persistence of these associations, even after adjusting for confounding factors and performing sex-stratified analyses, indicates that craniofacial pain, particularly in the temporal muscle, plays an important role in the manifestation of mental health disorders, with significant sex differences. These findings highlight the importance of a multidisciplinary approach in the diagnosis and treatment of TMD, considering not only biomechanical aspects but also the emotional components involved. Our results reinforce the relevance of incorporating mental health screening into dental care for patients presenting temporal pain complaints, especially women.

## Data Availability

The datasets generated during and/or analyzed during the current study are available from the corresponding author on reasonable request.
